# Intersectoral collaboration in zoonotic disease surveillance and response: A One Health study in the Greater Accra metropolitan area of Ghana

**DOI:** 10.1016/j.onehlt.2025.101137

**Published:** 2025-07-15

**Authors:** Joannishka K. Dsani, Sherry Ama Mawuko Johnson, Sandul Yasobant, Walter Bruchhausen

**Affiliations:** a One Health Graduate School, Center for Development Research (ZEF), University of Bonn, Genscherallee 3, 53113 Bonn, Germany; b Section Global Health, Institute for Hygiene and Public Health (IHPH), University Hospital Bonn, Venusberg Campus 1, Building 66, 53127 Bonn, Germany; c School of Veterinary Medicine, College of Basic and Applied Sciences, University of Ghana, P.O. Box LG139, Legon-Accra, Ghana; d Centre for One Health Education, Research and Development (COHERD), Indian Institute of Public Health Gandhinagar (IIPHG), Chiloda Road, Lekawada CRPF P.O. Gandhinagar, Gujarat 382042, India; e Department of Public Health Science, Indian Institute of Public Health Gandhinagar (IIPHG), Chiloda Road, Lekawada CRPF P.O. Gandhinagar, Gujarat 382042, India

**Keywords:** Zoonotic diseases, Surveillance, One health, Intersectoral collaboration, Health systems

## Abstract

The One Health (OH) approach is essential for preventing and managing zoonotic diseases through the promotion of intersectoral collaboration. Integrated surveillance systems enhance resource efficiency, support targeted interventions, and provide a comprehensive understanding of disease dynamics. However, operationalizing OH in resource-limited contexts encounters various obstacles due to systemic limitations. This study explores intersectoral collaboration across sectors engaged in zoonotic disease surveillance and response (ZDSR) in Ghana at the district level, identifying key operational gaps. We performed 46 key informant interviews with actors in the human, animal and wildlife health sectors directly involved in ZDSR. We developed an interview guide informed by WHO's *Components of Surveillance and Response Systems for Monitoring and Evaluation* Framework and the *Evaluation of collaboration in a multisectoral surveillance system* (*ECoSur)* tool. The responses were analyzed using a content analysis approach. The results showed that for district-level surveillance activities, relationships between the human and animal health sectors existed in 87.5 % of districts, albeit weak, while the wildlife sector was absent. Rabies/dog bites, Avian Influenza, Lassa fever and COVID-19 were the primary triggers of intersectoral collaboration. Anthrax, zoonotic tuberculosis and trypanosomiasis saw minimal intersectoral collaboration. Collaborative activities were mostly addressed through reactive and event-driven approaches focusing on case-specific data sharing, alerts in outbreak events, and committee participation. However, core surveillance functions like disease detection, data analysis, and data management practices were performed in siloes.

These results highlight the need for adaptable, proactive, and systemic frameworks that enhance intersectoral collaboration for the surveillance of neglected zoonotic diseases in Ghana.

## 1 Introduction

Zoonotic diseases cause about one billion illnesses and millions of deaths yearly posing significant threats to global health, livelihoods, and economies [1,2]. Effective zoonotic disease surveillance systems are critical for detecting, tracking, and controlling diseases, as evidenced by outbreaks of Ebola virus disease, Avian Influenza, and COVID-19 which is believed to have zoonotic origins [[3], [4], [5]].

The One Health (OH) approach is recommended to effectively prevent and control zoonoses by integrating human, animal, and environmental health sectors through intersectoral collaboration [6,7]. This approach supports better health outcomes and enhances disease surveillance by improving understanding of disease risks and fostering collaboration in detection and control efforts [6,8,9]. However, operationalizing OH in resource-constrained settings like that of sub-Saharan Africa faces significant challenges due to systemic limitations, institutional structures, and capacities [[10], [11], [12]]. Against these challenges, integrated approaches to disease surveillance and response remain essential.

Ghana is characterized by a high-risk environment for zoonoses [13] and prioritizes the OH approach, recognizing its important role in addressing health, economic, and livelihood implications. In 2017, Ghana's One Health Technical Working Group was established to draft legal frameworks for OH implementation [14]. The following year, a multi-stakeholder initiative used the CDC's One Health Zoonotic Disease Prioritization tool to rank 6 priority zoonotic diseases: (1) rabies, (1) Anthrax, (1) Avian Influenza, (4) zoonotic tuberculosis, (5) Viral Hemorrhagic Fevers (Lassa fever, Yellow fever, Dengue fever, Ebola) and (6) trypanosomiasis [15]. While these efforts demonstrate commitment to OH progress at the national level, they often do not translate into effective local-level operationalization, leaving their impact ambiguous in most attempts [16,17]. Limited integration of OH into surveillance systems hampers collaboration, necessitating understanding the dynamics of effective OH implementation.

This study sought to explore the current collaboration landscape primarily among the human health, animal health, and wildlife health sectors engaged in zoonotic disease surveillance and response (ZDSR), focusing on the practical application of the OH approach particularly at the operational (district) level. The outcome provides a comprehensive evaluation of intersectoral interactions, identifies operational gaps and offers actionable insights to enhance collaboration across sectors.

## 2 Methods

### 2.1 Study area and target population

The study was conducted in the Greater Accra Metropolitan Area (GAMA), Ghana's largest urbanized area with 25 districts [18,19]. In February 2022, during the proposal development phase, a stakeholder mapping exercise was conducted, including a workshop and individual interviews, to identify key institutions involved in ZDSR. The primary institutions identified were the Ghana Health Service (GHS) for human health, the Veterinary Services Department (VSD) for domestic animal health, and the Wildlife Division of the Ghana Forestry Commission for wildlife health. Representatives from the environmental health department under the Ministry of Local Government and Rural Development as well as the Environmental Protection Unit within the Ministry of Environment, Science, Technology, and Innovation (MESTI) indicated that their institutions lacked ZDSR systems. Thus, the study focused on the three sectors with established ZDSR systems.

The wildlife sector lacked district-level officers, as Ghana has only three wildlife veterinarians all of whom work at the national and regional levels. Consequently, the study focused on districts with both human and animal health sector representation. Of the 25 GAMA districts, four lacked animal health officers, leaving 21 districts eligible for inclusion. District-level authorities from GHS, VSD and the three wildlife veterinarians were contacted to schedule interviews, ensuring confidentiality and voluntary participation through informed consent. Participants were purposively sampled, targeting those directly involved with ZDSR and with over three months of experience in the district. Out of interest, one participant from a private hospital and a private veterinary clinic were included. Data for this study was collected as part of a larger study from December 2022 to December 2023 and analyzed between November 2023 and December 2024.

### 2.2 Data collection (Key Informant Interviews)

**Fig. 1:**
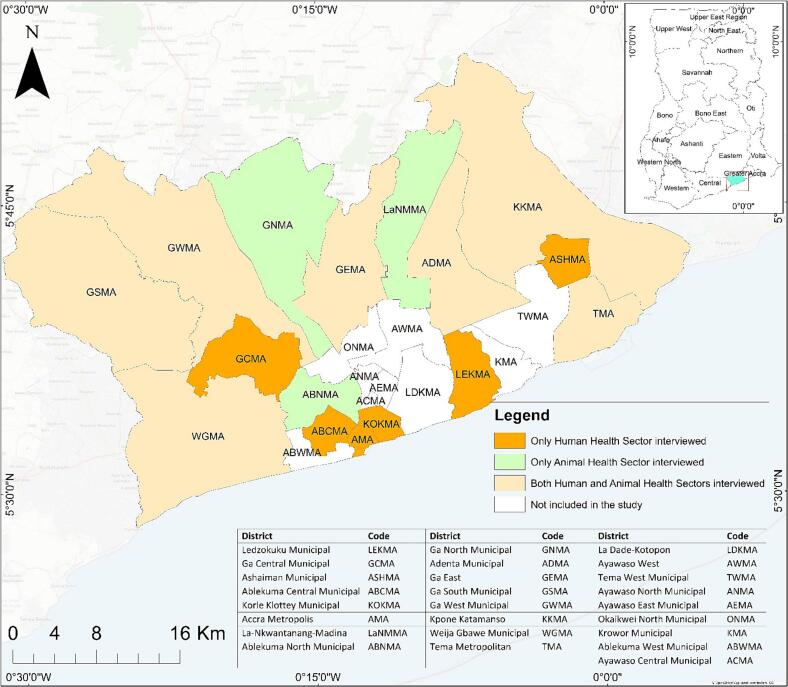
Map of the Greater Accra Metropolitan Area in Ghana, showing districts visited and sectoral representation from the human and animal health sectors.

This study defines intersectoral collaboration as any form of interaction between diverse actors, units, departments, agencies, and/or organizations of different sectors to enhance ZDSR at various levels. An interview guide was developed based on WHO's *Components of Surveillance and Response Systems for Monitoring and Evaluation* framework [20] which provides indicators for assessing communicable disease surveillance and response systems, and the *Evaluation of collaboration in a multisectoral surveillance system (ECoSur)* tool developed by Bordier et al. (2019) [21,22], which highlights governance and functional attributes necessary for collaboration in multisectoral systems (Appendix A). Open-ended questions were used to understand the nature of and rationale for sector interactions, the diseases driving collaborations, and the mechanisms through which the collaborations were implemented. Out of the 21 eligible districts, interviews were conducted in 16. Participants from both the human and animal health sectors were interviewed in seven districts, only the human health sector in six, and only the animal health sector in three (Fig. 1). These variations resulted solely from participant availability. All but two interviews were conducted by the 1st author, the remaining two by a trained research assistant. Audio recordings of the interviews, field notes, and copies of any collaboration-related documents deemed relevant by the participants were taken. The recordings were transcribed verbatim and pseudonymized before analysis.

### 2.3 Data analysis

The collected data were analyzed using a content analysis approach and coded using MAXQDA Analytics Pro 2022 software [23]. Transcribed interviews were thoroughly reviewed, and an initial coding framework was deductively developed using predefined categories. Additional codes emerged inductively through iterative data reviews. Themes were identified from the final code list and reorganized.

## 3 Results

A total of 46 key informant interviews (72 participants-40 females) were conducted within GAMA. Of these, 30 (65.2 %) were one-on-one, and 16 (34.8 %) involved groups of two to four participants representing the same sector. Among the participants, 46 (65.9 %) were from the human health sector (HH), 23 (39.9 %) from the animal health sector (AH), and 3 (4.2 %) from the wildlife sector (WI). Key informant interviews were conducted at the district level (86.1 %), regional (2.8 %) and national levels (9.8 %). During analysis, all 46 interviews representing 16 districts were synthesized into 25 sector profiles: 13 sources of information (SOI) for the human health sector, 10 for animal health, and two for wildlife.

The results were categorized into 4 primary groups (1) **Who** – identifying the sectors involved in intersectoral collaborations; (2) **What** – examining the diseases driving these collaborations; (3) **Where** – analyzing the specific areas of surveillance and response where these interactions occur; and (4) **How -** identifying the legal frameworks that enable and guide these collaborations.

### 3.1 Who - sector and actor interactions

**Fig. 2:**
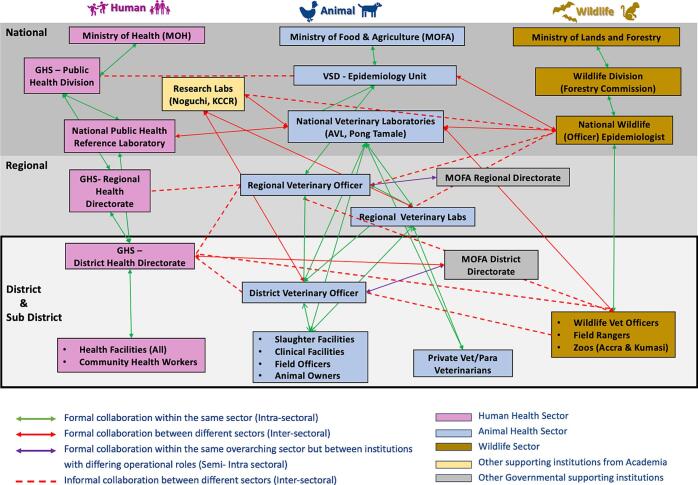
Mapping of Interactions Among Key Actors in Zoonotic Disease Surveillance and Response in the Greater Accra Metropolitan Area of Ghana. This diagram illustrates the operational structure and collaborative linkages between human, animal, and wildlife health sectors in zoonotic disease surveillance across the national, regional, and district levels in Ghana, based on participant interviews. **Green solid lines** represent ***intra-sectoral collaboration***, showing formal, structured linkages within a single sector. **Red solid lines** denote ***inter-sectoral collaboration***, indicating formal collaborations between distinct sectors. **Violet solid lines** indicate ***semi-intra sectoral collaboration***, representing formal collaboration within the same overarching sector but involving institutions with distinct operational roles. **Red dashed lines** indicate ***informal intersectoral collaboration***, often based on personal communication or ad hoc coordination rather than formal frameworks. The human health sector is represented in pink**,** the animal health sector in blue and the wildlife sector in brown. Academic research laboratories are shown in yellow, while supporting governmental institutions are represented in grey**.**

Fig. 2 illustrates the mapping of key actors involved in ZDSR in GAMA, as identified from participant interviews.

#### 3.1.1 Interactions between main OH sectors (human, animal, and wildlife) only

Of the three participating sectors, bilateral interactions were only between the human and animal health sectors. Of the 16 districts, 37.5 % of human and animal health sectors showed strong intersectoral relationships, 50 % exhibited weak ones, and 12.5 % had none. These classifications were based on the number, strength, and frequency of collaborative activities, evidence of initiative from both sectors and the ability of actors to identify colleagues from other sectors. Collaboration analysis revealed that in 36 % of districts, both sectors co-led initiatives, 21 % were primarily human health-driven, 7 % were animal health-driven, and in the remaining 36 %, the lead sector was unclear. Across all sectors, most collaborative activities were either one-time occurrences or infrequent.

Collaborations between the wildlife sector and others were one-sided, as none of the human or animal health SOIs mentioned it. We observed that some participants from the human health sector appeared visibly perplexed when asked about previous collaboration with the wildlife sector. This seemed to stem from uncertainty about the relevance of wildlife collaboration to their district-level responsibilities, suggesting they had difficulty conceptualizing how such collaboration**s** would apply to their scope of practice. This reaction contrasted sharply with their responses about collaborations with the animal health sector, which were more familiar and expected. Wildlife SOIs provided limited examples of intersectoral collaboration nationwide. In GAMA, these included a one-time joint Brucellosis sampling exercise witnessed by the research team between zoo staff and GHS staff of a non-participating district, sporadic disease reporting to VSD and sample testing at veterinary laboratories. Other examples were minimal and either occurred outside GAMA, were unrelated to ZDSR, or did not involve district-level collaborations.

**Fig. 3:**
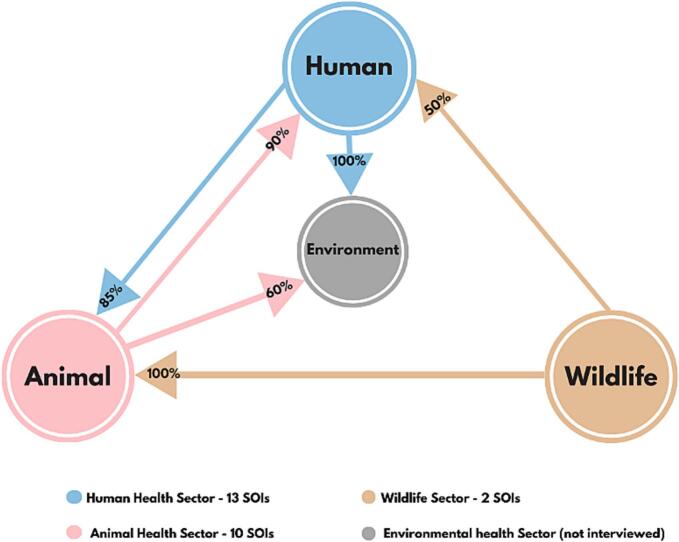
The percentage breakdown of sector-to-sector interactions between the main One Health sectors and the environment health sector in the Greater Accra Metropolitan Area (GAMA).

Despite the environmental health sector's exclusion as a key institution based on previous stakeholder feedback, it emerged repeatedly during interviews. This was particularly evident in the frequent collaborations between the human health sector and the District Environmental Health and Sanitation Department, reported by 100 % of human health SOIs. Human health actors attributed this to clear legislative mandates guiding such collaborations. Although 60 % of animal health SOIs reported collaborating with the environmental sector, their primary collaboration partner remained the human health sector. Given the environmental sector's significant role, it was included in the results visualization (Fig. 3).

#### 3.1.2 Interactions between the main OH sectors and other supporting sectors

Multisectoral collaborations spanning the environmental health department, media, security, education, academia, non-governmental organizations (NGOs), and governmental agencies were mentioned by 22 of the 25 SOIs. Examples included joint vaccination campaigns, joint education efforts and district assembly-facilitated committees. Participants were engaged in joint social media platforms, joint sampling, outbreak investigations, and training.

In analyzing one-on-one collaborators, district environment departments (HH-100 %, AH-60 % WI-0 %) and district assemblies (HH-85 %, AH-50 % WI-0 %) were mentioned the most. For the animal health sector, the district agriculture department was the primary collaborator (HH-46 %, AH-80 %, WI-0 %). While veterinary officers are technical authorities for animal health, their administrative placement under district agriculture directors created confusion during the interviews with some human health SOIs conflating the ‘animal health sector’ with the ‘agriculture department’. This structural overlap led to uncertainty regarding representation on health committees, which some veterinary officers acknowledged, sometimes excluded them from intersectoral spaces even when zoonoses were discussed.

Other, supporting sectors mentioned included education (HH-85 %, AH-40 %, WI-0 %), security (HH-54 %, AH-50 %, WI-0 %), academia/research (HH-0 %, AH-0 %, WI-50 %), other governmental agencies like the National Disaster Management Organization (NADMO) (HH-39 %, AH-60 %, WI-0 %), media (HH-54 %, AH-10 %, WI-0 %), and NGOs (HH-23 %, AH-30 %, WI-0 %).

#### 3.1.3 Private sector collaborations

Of the 46 interviews, only 5 reported collaborations with private actors. All examples were within the same sector and restricted to disease reporting only. No examples of cross-sector collaborations, such as between private animal health facilities and government human health facilities, were noted. Private sector interviews from both sectors revealed a primary focus on healthcare delivery. Private human health actors stated all suspicious cases were routinely reported to public health authorities whereas private animal health actors referred cases through clients to public animal health authorities.

### 3.2 What – diseases as drivers/triggers of collaboration

Based on the combined weighted average, rabies and/or dog bites were the main drivers/triggers of intersectoral collaborations (HH-85 %, AH-80 %, WI-0 %) across all three sectors. Avian Influenza was the second highest driver (HH-54 %, AH-90 %, WI-0 %) and was mainly addressed through multisectoral efforts led by the animal health sector involving NADMO and occasionally, the environmental and human health sectors. Other priority zoonoses of Ghana like trypanosomiasis, zoonotic tuberculosis, Dengue fever, and Anthrax had little to no mention. Some human health participants suggested that the lack of collaboration on tuberculosis could stem from district-level diagnostics not confirming zoonotic origins, and treatment protocols remained unchanged regardless of origin making collaboration seem unnecessary and unclear. Other explanations included the absence of cases in participating districts (Anthrax) or nationally (Ebola, Dengue fever) and uncertainty about their sector's role in the control of a particular disease (animal health on Yellow fever and Lassa fever). No rationale was provided for trypanosomiasis. COVID-19 (a non-priority zoonosis) and Lassa fever accounted for 44 % and 40 % of mentions, respectively. However, COVID-19 was mentioned only by the human health sector as a driver of collaboration with the environmental health department and other support sectors during outbreak responses. See Table 1 for further details.

**Table 1:** Diseases that triggered intersectoral collaboration in the greater accra metropolitan area and the percentage of participant sources mentioning them.

	**Diseases**	**Human health** **(*n*** **= 13)**	**Animal health** **(*n*** **=** **10)**	**Wildlife** **(*n*** **=** **2)**	**Weighted total average (%)** **(*n*** **=** **25)**
1	Rabies	85 %	80 %	0	76 %
2	Anthrax	0	0	0	0 %
3	Avian Influenza	54 %	90 %	0	64 %
4	Zoonotic Tuberculosis	8 %	10 %	0	8 %
5	Trypanosomiasis	0	0	0	0 %
6a	Ebola	8 %	0	0	4 %
6b	Yellow Fever	15 %	0	0	8 %
6c	Lassa Fever	54 %	30 %	0	40 %
6d	Dengue Fever	0	0	0	0 %
7	COVID-19	85 %	0	0	44 %
8	Mpox	0	10 %	0	8 %
9	Brucellosis	0	0	50 %	4 %

The 6 prioritized zoonotic diseases (1-6d) are listed as ranked in FAO Ghana 2018 [15] followed by non-priority zoonoses (7–9) mentioned by participants.

### 3.3 Where – Areas of collaboration

The areas of how collaboration occurred are presented in Table 2. Results were categorized into technical functions, communication & data sharing mechanisms, coordination, dissemination, and ‘others’. The most frequently (68 % SOIs) mentioned collaborative activity was ‘data sharing on specific cases.’ Other high-ranking areas included issuing ‘alerts,’ membership in intersectoral committees, and joint outbreak responses. Public education ranked high, but examples of collaborations were primarily limited to human health and education sectors. Participants provided examples of collaborations they believed fell under disease detection, registration, confirmation, and data analysis. However, analysis revealed these were intra-sectoral rather than intersectoral activities.

**Table 2:** Areas of intersectoral collaboration in zoonotic disease surveillance and response in the greater accra metropolitan area of ghana with percentage of participant sources mentioning them.

**Areas of collaboration**	**Sub-areas of collaboration**	**Human** **Health** **(*n*** **=** **13)**	**Animal** **Health** **(*n*** **=** **10)**	**Wildlife** **(*n*** **=** **2)**	**Weighted Total** **Average** **(%)**
**Technical Functions**	Disease Detection	0	0	0	0
	Disease confirmation	0	0	0	0
	Data Analysis & Interpretation	0	0	0	0
	Response	46 %	50 %	100 %	52 %
	Control	23 %	60 %	0	36 %
**Communication &** **Data Sharing Mechanisms**	Alerts	77 %	60 %	0	64 %
	Routine Reporting	46 %	40 %	50 %	44 %
	Other communicative mechanisms/platforms	62 %	20 %	0	40 %
	Data Sharing - Routine	23 %	30 %	50 %	28 %
	Data sharing - specific cases (mostly one-time)	85 %	60 %	0	68 %
	Data management	0	0	0	0
**Coordination**	Committees' membership	77 %	50 %	0	60 %
	Other meetings	31 %	50 %	0	36 %
	Coordination – program/activity	15 %	20 %	0	16 %
**Dissemination**	Public Education	92 %	20 %	0	56 %
**Others**	Training	15 %	60 %	100 %	40 %
	Research	0	20 %	50 %	12 %
	Legislation – SOP formulation	15 %	10 %	0	12 %
	Resource sharing	15 %	20 %	0	16 %
	Advocacy	15 %	0	0	8 %
	Planning	0	0	0	0

### 3.4 How – Documentation

Participants were asked whether their reported collaborations were formal or informal. While most acknowledged that many collaborative activities were informal, driven by individual initiative and pre-service training, others were believed to have legal backing. For example, human health sector participants cited the organization and composition of health committees as formal collaborations supported by the Technical Guidelines for Integrated Disease Surveillance and Response in Ghana (IDSR 3rd edition) and the Local Governance Act 2016. A review of the IDSR supported this assertion, but the Local Governance Act did not. Another document mentioned was the Public Health Act, 2012 (Act 851), although a more in-depth analysis of all documents was beyond the scope of this study.

The wildlife sector indicated that wildlife health issues were managed according to VSD's policies. Both animal health and wildlife officers cited the ‘yellow book’ (Animal Disease Surveillance Guide of VSD) as their primary reference, but a review revealed no directives on collaboration within this document. Additional documentation mentioned included dog bite case management forms created independently by some animal health participants and Avian Influenza destruction forms issued by VSD, requiring signatures from multiple actors such as NADMO, the environmental department and the district assembly.

## 4 Discussion

This study investigated the current state of intersectoral collaboration in ZDSR in GAMA, identifying key actors, diseases driving collaboration, and functional interactions. Unlike previous studies focusing on single-disease systems, this research broadens the scope by examining the same actors collaborating in a more extensive integrated health system and quantifying the intersectoral activities. The key findings highlight existing but weak relationships between human and animal health sectors in most of the districts. The wildlife sector was largely absent at the district and sub-district level, while human health and environment sectors maintained a strong, legislatively supported relationship. Collaborative efforts were largely limited to activities such as committee participation, outbreak alerts during outbreaks, and case-specific data sharing, while core functions like disease detection and data management were carried out in isolation. Diseases driving collaborations were Lassa fever, Avian Influenza, rabies/dog bites, and COVID-19, with little collaboration observed for other priority zoonoses. To our knowledge, it is the first study in Ghana to analyze collaborative activities across multiple surveillance domains and assess the extent and nature of these interactions.

### 4.1 Sectoral gaps and collaboration barriers

The lack of the wildlife sector at the district/sub-district levels constitutes an important gap in the OH Approach, thereby compromising comprehensive disease surveillance and response [24,25]. This exclusion creates blind spots in surveillance, inhibits knowledge-sharing, and weakens the overall capacity to effectively prevent and control zoonotic diseases [25]. Wildlife surveillance remains important for disease transmission even in highly urbanized areas like GAMA. Besides the Accra Zoo, GAMA is home to two wildlife-protected wetlands: the Sakumo and Densu Delta Lagoons [26]. The Shai Hills Resource Reserve wildlife park is also located near GAMA [26]. Moreover, the trading and consumption of wildlife such as large rodents (e.g., grasscutters - *Thryonomys swinderianus*), along with the presence of urban-dwelling animals like bats, common rodents (e.g., rats) and avian scavengers (e.g., vultures and crows) can significantly contribute to disease spread [[27], [28], [29], [30]]. Given limited financial and human resources, it is understandable that wildlife surveillance efforts may prioritize areas outside GAMA with higher wildlife populations and biodiversity, leaving urban areas with insufficient coverage [11,24]. The findings in GAMA, which indicate no intersectoral collaborations with the wildlife sector, an absence of district-level legislative frameworks and an extremely limited wildlife workforce, lead to fragmented systems and little data sharing [25]. This neglect, seen globally, reflects broader gaps in policy and strategic planning, leading to chronic underinvestment and insufficient political support for integrated, multisectoral approaches [[31], [32], [33]]. Research indicates that countries conducting wildlife surveillance detect zoonotic diseases earlier and respond faster [6,25]. However, outside specific outbreaks, the wildlife sector is still perceived as playing an insignificant role or making no substantial contribution [33]. Additionally, the weak relationship between the human and animal health sectors in GAMA, compounded by administrative and legislative deficiencies, further hinders effective collaboration [31]. Addressing these issues requires integrating the wildlife sector into disease surveillance and fostering stronger collaborations across sectors to establish a more cohesive and effective response framework [31].

### 4.2 Nature and effectiveness of current collaborations

Another key finding was the reactive and event-driven nature of intersectoral collaborations, indicating a broader systemic issue of unsustainable and inconsistent practices [34]. For instance, ‘outbreak alerts’ and ‘data sharing on priority cases’ were common practices, whereas routine resource-intensive activities necessitating sustained, active effort often fizzled out after initial attempts. Additionally, collaboration seemed to favor easier, less demanding activities like committee meeting participation. This reactive approach, where collaborations are mobilized only during crises, limits long-term readiness and prevents the establishment of proactive frameworks under the OH Approach [35,36]. Without clear protocols or mechanisms for intersectoral collaboration, such initiatives often decline over time [[37], [38], [39]].

Diseases with immediate or visible impacts such as rabies, and those with pandemic potential including Avian Influenza, Lassa fever, and COVID-19, a non-priority zoonosis triggered coordinated efforts. Contrastingly, less visible, endemic but priority diseases such as trypanosomiasis and zoonotic tuberculosis were overlooked due to the absence of cases, diagnostic limitations, unclear sectoral roles, or insufficient multisectoral coordination frameworks. Other studies confirm that zoonotic disease prioritization varied based on factors similar to those identified in this study with greater policy attention given to globally recognized zoonoses than to low-profile or endemic ones that pose long-term threats [33].

Addressing these challenges requires a shift from event-driven to systematic collaboration within the OH Approach. Proactive and sustainable collaborative frameworks must prioritize routine communication, role clarification, accountability, diagnostic improvements, cross-sectoral surveillance, capacity-building in neglected diseases, resource allocation, and legislative support [40]. Such initiatives foster consistent, effective collaboration and integrated OH strategies prioritizing immediate threats and long-term public health resilience. Effective OH collaboration should be proactive and adaptable to different disease contexts [40].

### 4.3 Limitations

This study offers useful insights into the dynamics of intersectoral collaboration under the OH Approach but is not without limitations.

#### 4.3.1 Interview bias

We realized that the picture of collaboration within districts could vary depending on the informant. Additionally, in districts where only one sector was represented, there was the possibility of obtaining an incomplete picture. To mitigate these, we prioritized interviewing senior and long-serving surveillance actors with extensive institutional knowledge. We also cross-validated data across sectors in the same district wherever possible. Of the 16 districts visited, cross-validation was feasible in 7, with inconsistencies detected in only one district. This discrepancy revealed a significant oversight: human health actors had unknowingly been collaborating with a subordinate of the animal health authority instead of the designated lead. Notably, this study facilitated the identification and correction of this gap.

#### 4.3.2 Recall bias

Participants' ability to recall events could affect the comprehensiveness of the information provided. A thorough, pilot-tested interview guide was used and supplemented with clarifications and probing during interviews to reduce this. Participants were encouraged to share any additional information they recalled post-interview.

#### 4.3.3 Geographic and temporal bias

The study was conducted during recent outbreaks of Lassa fever [41], Avian Influenza [42,43], and COVID-19 [44,45] which could indicate short-term interests that may not be indicative of the reality long-term. However, this study gives valuable insights into how collaborations occur during crises, even if they may not fully represent long-term realities.

## 5 Conclusion

This study reveals significant structural and legislative barriers to effective intersectoral collaboration. It underscores the absence of the wildlife sector in surveillance and response activities and highlights the reactive, event-driven nature of existing intersectoral collaborations. The findings point to the need for more adaptable, proactive, and systemic frameworks to enhance collaboration for neglected zoonotic diseases. By focusing on improving diagnostic capabilities, clarifying sectoral roles, and streamlining legislative and administrative frameworks, ZDSR systems in Ghana can become more cohesive, efficient and effective in managing zoonotic diseases and enhancing public health outcomes. Future research should adopt longitudinal designs, expand geographic and sectoral coverage, and incorporate standardized metrics to assess the sustainability and effectiveness of collaborations.

## Funding sources

This work was supported by the Ministry of Culture and Science of North Rhine-Westphalia, Germany through the grant *Forschungskolleg ‘One Health and Urban Transformation.’* The work was also supported by a travel grant awarded by the The Soulsby Foundation for One Health, United Kingdom (https://soulsbyfoundation.org). None of the funding bodies were involved in the design of the study, collection, analysis, and interpretation of data in writing the manuscript.

## Data statement

Data from this study is available at the One Health Graduate School, Center for Development Research (ZEF), University of Bonn, Bonn, Germany. Researchers who meet the criteria for access to confidential data can contact the Coordinator, Fortschrittskolleg ‘One Health’, Center for Development Research (ZEF), Bonn, Genscherallee 3, 53113 Bonn, Germany. Email: health@uni-bonn.de

## Declaration of generative AI and AI-assisted technologies in the writing process

During the preparation of this work, the author used Quillbot and Grammarly to correct grammar and improve the readability of the manuscript. After using this tool/service, the author reviewed and edited the content as needed and takes full responsibility for the content of the published article.

## Disclosure statement

The authors declare that they have no competing interests.

## Ethics approval

Before the study, ethical clearance and formal permissions were obtained from the University of Bonn – Centre for Development Research Ethics Board (22c_Joannishka Dsani), the University of Ghana – Ethics Committee for the Humanities (ECH 061/22–23), the Ghana Health Service Ethics Review Committee (GHS-ERC: 023/09/22), the Veterinary Services Department of the Ministry of Food and Agriculture and the Wildlife Department of the Forestry Commission of Ghana.

## CRediT authorship contribution statement

**Joannishka K. Dsani:** Writing – review & editing, Writing – original draft, Visualization, Project administration, Methodology, Investigation, Funding acquisition, Formal analysis, Conceptualization. **Sherry Ama Mawuko Johnson:** Writing – review & editing, Supervision, Methodology, Conceptualization. **Sandul Yasobant:** Writing – review & editing, Supervision, Methodology. **Walter Bruchhausen:** Writing – review & editing, Supervision, Resources, Methodology, Funding acquisition.

## Declaration of competing interest

The authors declare the following financial interests/personal relationships which may be considered as potential competing interests:

Joannishka K. Dsani reports financial support was provided by The Soulsby Foundation for One Health. If there are other authors, they declare that they have no known competing financial interests or personal relationships that could have appeared to influence the work reported in this paper.

## Data Availability

Data will be made available on request.

## Glossary

AH: Animal Health Sector
COVID-19: Coronavirus disease 2019
ECOSUR: Evaluation of collaboration in a multisectoral surveillance system tool
FAO: Food and Agriculture Organization of the United Nations
GAMA: Greater Accra Metropolitan Area
GHS: Ghana Health Service
HH: Human Health Sector
MOFA: Ministry of Food and Agriculture
MOH: Ministry of Health
NADMO: National Disaster Management Organization, Ghana
NGO: Non- Governmental Organization
OH: One Health
SOI: Sources of Information
WHO: World Health Organization
WI: Wildlife Sector
VSD: Veterinary Services Department
ZDSR: Zoonotic Disease Surveillance and Response

## Appendix A Supplementary data

Supplementary material Image 1
