# Supplementary material for: Intersectoral collaboration in zoonotic disease surveillance and response: A One Health study in the Greater Accra metropolitan area of Ghana
Source: One Health. 2025 Jul 15;21:101137. doi: 10.1016/j.onehlt.2025.101137 (PMC12284660; doi:10.1016/j.onehlt.2025.101137)
Supplement: Supplementary file 1 — Supplementary material [file mmc1.pdf]

Appendix A – Supplementary Data

Interview Guide for Intersectoral Collaboration Assessment in the Surveillance and Control of Zoonotic Diseases (For Interviewer)

Part 1: Introduction

1. An introduction to the research and its team.
2. Participants read the participant information sheet and ask questions/seek clarifications.
3. Obtain permission from participants to participate in an interview, audio record the interviews and if required, take pictures.
4. Participants sign the consent form.
5. Participants fill out demographic data form.

Part 2:

Topic: Intersectoral Collaboration with Other Sectors Other Than Your Own

1. When it comes to zoonotic disease surveillance and response activities, do you collaborate with other actors (offices, departments, staff) from other sectors? If yes, with whom and how?
    a. *Probe for explicit descriptions and details.*
    b. *Note down the examples so that you can refer participants to them for follow-up questions.*

c. *Prompts: human health, animal health, environment, wildlife, media, education, security services, NGOs, the assembly, etc. Include any other new actor/sector mentioned in subsequent interviews*

2. In which areas of surveillance do you collaborate with other sectors?
    a. *For each area, ensure that participants are only referring to district/subdistrict level collaborations as some participants work at various levels and may conflate the activities.*
    b. *Use the following as prompts to trigger participants' memories.*
        i. *Coordination - e.g., of persons, units, departments, agencies, committees of an outbreak response involving other sectors.*
        ii. *Surveillance technical functions – e.g., joint activities related to outbreak detection, response, control*
        iii. *Scientific support– e.g., seeking technical advice from another sector, conducting research together, etc.*
        iv. *Protocol design – e.g., joint activities related to the designing SOPs, job aids, etc.*
        v. *Sampling activities - e.g., joint activities related to the taking, packaging or transporting of human/animal samples*
        vi. *Laboratory activities - e.g., joint activities involving any laboratory of another sector (case confirmations)*
        vii. *Data analysis and interpretation – joint activities related to the analysis and/or interpretation of another sector's data*
        viii. *Data management and storage – joint activities related to the storage and/or management of surveillance data*

ix. *Exchange/Reporting of surveillance data (routine) – activities involving sharing or receiving surveillance data to or from another sector. Take note of the frequency.*

x. *Communication - e.g., modes, frequency*

xi. *Dissemination - e.g., events, joint visits to media platforms to share zoonoses, collaborative visits to schools, etc.*

xii. *Other collaborative areas - e.g., trainings, meetings, planning, resource sharing etc.*

3. Is there any other form of collaboration you have had with another sector that was not triggered by a disease outbreak?
4. Inquire about more collaborations, using the diseases (suspected or confirmed) as prompts:
   a. Have you had a ……. case before?......... If yes, did you collaborate with any sector during that time? *(Prompts: Rabies/Dog bite, Anthrax, Avian Influenza, Zoonotic Tuberculosis, Lassa fever, Ebola Virus Disease, Yellow fever, Dengue Fever, Trypanosomiasis).*
   b. Is there any other disease that I have not mentioned yet where you worked with another sector?
5. Which of the collaborations that you mentioned are formally written in a document?
   a. *Take note of the collaborative area and the specific document name.* (*If the participant has a copy of said document, note down the name or take pictures where possible.*)
   b. *Refer participants to your notes, should they forget any examples given.*
6. (*If there are examples of formal collaborations given*) Are you implementing these formal collaborations as they should? If not, why?

7. Which of the collaborations that you mentioned are informal (please list)?
    a. *Refer participants to your notes, should they forget any examples given.*
8. Do you have any other comments?

   (Prompt participants to reach out if they remember any important information post-interview.)

THANK YOU VERY MUCH FOR YOUR TIME!
